# A Compressive Armchair (OTO) to Perform Deep Pressure Therapy in Children With Autism Spectrum Disorder: User-Centered Design and Feasibility Study

**DOI:** 10.2196/55754

**Published:** 2024-11-05

**Authors:** Thomas Gargot, Amandine Vachaud, Clémence Gilard, Alexia Audrain, Marie Gomot, Marco Guidotti, Frédéric Briend, Joëlle Malvy, Frédérique Bonnet Brilhault

**Affiliations:** 1 Child and Adolescent Psychiatry Department University Hospital Tours Tours France; 2 EXcellence Center in Autism and Neurodevelopmental Disorders—Tours ExAC-T Tours France; 3 Université de Tours INSERM Imaging Brain & Neuropsychiatry iBraiN U1253, 37032 Tours France; 4 Labaa Company Nantes France

**Keywords:** deep pressure therapy, proprioception, compression, autism spectrum disorder

## Abstract

**Background:**

Deep pressure therapy (DPT) is widely used to reduce anxiety in children with autism spectrum disorder (ASD), but evidence of its efficacy is limited.

**Objective:**

This study aims to design a usable, nonstigmatizing compressive armchair that can be easily controlled, electronically, by the user.

**Methods:**

A user-centered approach was used to assess the usability of the device. Testing was carried out in a day hospital for children with ASD in France, with a convenience sample of children with severe forms of ASD and intellectual deficiency (N=39). The Witteman design guideline was used. The System Usability Scale and time of use were reported.

**Results:**

The final product is a compressive armchair designed to be user centered, with 4 different cells that can be inflated to induce tailored pressure on the body. The pressure level is recorded electronically. Usability was between good and excellent. The device was used by 39 children, once or twice weekly, over a period of 31 months. Each session lasted between 3 and 20 minutes. The armchair takes up less space than a hug machine. Performing sessions with the chair is feasible.

**Conclusions:**

First clinical impressions show a decrease in anxiety, improved emotional regulation, and improved attention. DPT is widely used in occupational therapy and frequently requested by parents, but efficacy studies are too scarce to make evidence-based recommendations for its use. The results presented here support further controlled efficacy studies of DPT in the treatment of anxiety in children with ASD.

## Introduction

### Background

Autism spectrum disorder (ASD) is defined by (1) persistent deficits in social communication and social interaction across multiple contexts and (2) restricted, repetitive patterns of behavior, interests, or activities [1]. According to the American Psychiatric Association [1], the prevalence of autism is 1%.

Sensory difficulties are frequently found in individuals with ASD sensitivity [2], in particular somatosensory system difficulties such as aberrant skin sensitivity [3,4] (including pressure detection) and proprioception. These sensory anomalies could underlie the pathophysiological processes that lead to impaired social development [5].

Proprioception is the sensory registration of the ongoing spatial configuration of the body. It includes the position of the body segments in space, the force and the speed of movement, and the integration of gravity and body balance. Proprioception impacts behavioral regulation and motor control [6]. Blanche et al [7] showed that children with ASD present proprioceptive processing difficulties that are different from those of children with other developmental disabilities and their typically developing counterparts. However, Morris et al [8] and Fuentes et al [9] did not confirm these proprioceptive difficulties in experimental paradigms. It is possible that the deficits rely mostly on multisensory integration [10].

### Sensory Integration Theory

Ayres et al [6] introduced sensory integration theory to explain sensory processing issues in children with ASD. The focus of sensory-based intervention is to maintain an optimal level of arousal between hypo- and hyperstimulation, allowing the individual to respond to the environment in an adaptive manner [11,12].

Several techniques or devices can be used in sensory-based interventions, in particular deep pressure therapy (DPT); for instance, Wilbargers suggested a deep pressure and proprioceptive protocol [13]. Systematic reviews show that sensory integration therapies, which use play activities and sensory-enhanced interactions, have positive effects, but the quality of the studies is not sufficient to confirm these results [11,14]. Nevertheless, the American Occupational Therapy Association recommends the use of sensory strategies for individuals with ASD [12,14].

In a web survey involving 552 parents of children with ASD, Green et al [15] showed that sensory integration was the third most used treatment after speech therapy and visual schedules and before behavioral methods. In a survey involving 152 parents of children with ASD, Peña et al [16] reported high acceptability of sensory-based methods. These interventions were considered to be “very important” or “important.” Main barriers to the use of sensory-based methods were the lack of recommendations and difficulty in using or difficulty in accessing this kind of intervention [16]. Among these techniques, DPT was of particular interest.

### Devices and Strategies for DPT

Different devices and strategies can be used to deliver DPT to patients with ASD (Table 1).

**Table 1:** Comparison of several devices used to induce deep pressure therapy in children with autism spectrum disorder.

Type of device	Principle	Level of evidence	N^a^	Time of use	Measure of efficacy	Control	Efficacy	Acceptance	Cost	Autonomy of the patient	References
Weighted blankets	Weight	Systematic review, population-based observational study	Observational: 1785; interventional: 160	>8 h daily	Sleep, STAI^b^, electrodermal activity, pulse rate	Nothing or light plastic chain blanket	Conflicting evidence	+++^c^	+	+++	[17-25]
Therapeutic body wrap	Tightening	One RCT^d^	48	45 min; 2 times/wk	Aberrant Behavior Checklist, irritability	Dry vs wet sheet therapeutic body wrap	+ but no waiting list comparative arm	+/–	+	– – –	[26-29]
Shape memory vest	Tightening	None, prototypes	None	Unknown	None	None	Unknown	Unknown	++	Unknown	[30,31]
Compression vest	Pressure by inflation	SCRD^e^	3	20 min, daily (unclear) for 22-50 d	Stereotypies	Fully deflated vest or no vest	No efficacy	+++ ?	++	+++	[32,33]
Manual squeezing	Manual squeezing	SCRD	8	5-15 min, until 3 times/d for 3 mo	Visual analog scales (calmness, engaged, responsivity, happy, communicative)	None	+/–	+++	+	–	[34]
Hug or squeeze machine	Compression by a plate	RCT	12	20 min; 1 time/wk for 6 wk	The Conners Parent Rating Scale, electrodermal activity	Not receiving deep pressure in the disengaged hug machine	++	+	+++ (reusable)	–	[35-40]
Compressive garments	Tightening	Observational study	14	>1-16 h daily for 6 wk	Aberrant Behavior Checklist, sensory integration (Dunn Sensory. Profile), postural sway, motor performance	None	+ but no comparative arm	+++	+++ (tailored)	+/–	[6]
Sitting hug machine	Compression by a plate	SCRD	2	Not reported	Stereotypical behaviors	None	+ but no comparative arm	+++	++ (reusable)	+++	[35]
Compressive chair	Compression by inflated cushions	None, prototype	None	Unknown	None	None	Unknown	+++	++ (reusable)	+++	—^f^

^a^N: number of included studies in a systematic review.

^b^STAI: State Trait Anxiety Inventory-10.

^c^+++ (very) to - - - (not at all).

^d^RCT: randomized control trial.

^e^SCRD: single case research design.

^f^Not applicable.

### Hug or Squeeze Machine

Krauss [35] used a pressure apparatus consisting of 2 stacked air mattresses to deliver DPT to 23 typically developing college students during an examination period. Heart rate and self-reported anxiety were measured using the State-Trait Anxiety Inventory. The control group did not receive DPT. There was no objective difference between the 2 conditions. Subjectively, participants in the deep pressure group reported a relaxing effect. The baseline level of anxiety in this population was low. The author did not exclude that the confinement alone may have induced subjective feelings of relaxation.

Grandin [41] developed a hug device to allow self-administration of lateral body pressure for individuals with high levels of anxiety. Edelson et al [36] tested this device on children with ASD (n=12). A control group received deep pressure via a disengaged hug machine. Participants had 20-minute sessions every week for 6 weeks. Arousal and anxiety was measured using the Conners Parent Rating Scale and electrodermal activity. The hug device decreased anxiety according to both behavioral and physiological measures. As pressure can be controlled by the individual, this device may be useful for children with marked anxiety. No side effects were reported.

The ergonomics of the device are an important issue [36,42]. Lo and Huang [38] interviewed professionals and patients, concluding that it was important to improve the ergonomic design of the device and its acceptability. For instance, it is necessary to lie down or to squat, which can be difficult for some children. The system is very bulky. The controller is outside of the machine and cannot be activated autonomously. It is not possible to choose the part of the body that the individual or professional wants to squeeze.

Lo and Huang [38] suggested a sitting hug machine that is more compact, controllable by the patient or the therapist, and can apply pressure selectively to either the shoulders or bottom part of the body. Stereotypies decreased during intervention for 2 children.

Afif et al [39] designed a portable, inflatable hug machine. It was tested on 5 children with ASD. They measured heart rate variability. They found that the inflatable wrap model decreased heart rate but could not find this effect with a manual pull [40].

This paper reports the design of a hug machine that aimed to (1) improve controllability of the pressure by the professional, allowing replicability and making the device useful for both care and research; (2) improve controllability of the pressure by the children or adults with ASD, allowing different pressure on the bottom and top of the body; (3) use pressure instead of restraint; and (4) be more attractive and less stigmatizing. This paper describes the design method and the device itself and evaluates the usability of the device.

## Methods

### Overview

In a population with special needs such as ASD with intellectual deficiency (ID), gathering children or adults with ASD feedback could be complicated by communication and social difficulties. It is important to have a tailored, user-centered strategy to improve acceptability and usability before assessing efficacy. Because many of the children were not verbal due to associated ID, we collected feedback from the professionals who guided and observed the children during the care. We also asked feedback from 1 adult with ASD. The first uses of the device were video recorded to tailor the design of the device to the needs of the patient and the therapist.

### Design Goal and Process

#### Overview

**Figure 1:**
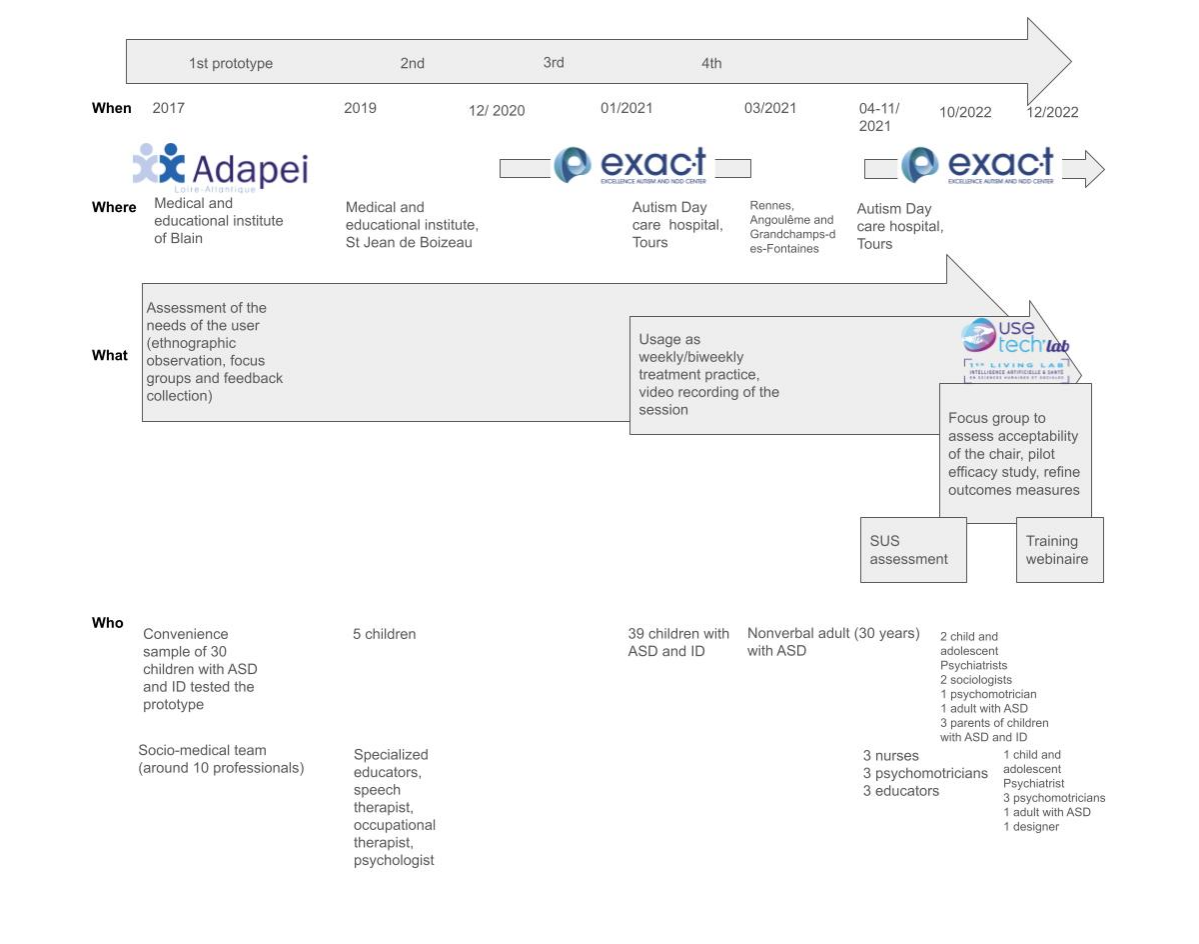
Timeline of the design of the OTO chair. ASD: autism spectrum disorder; ID: intellectual deficiency; SUS: System Usability Scale.

**Figure 2:**
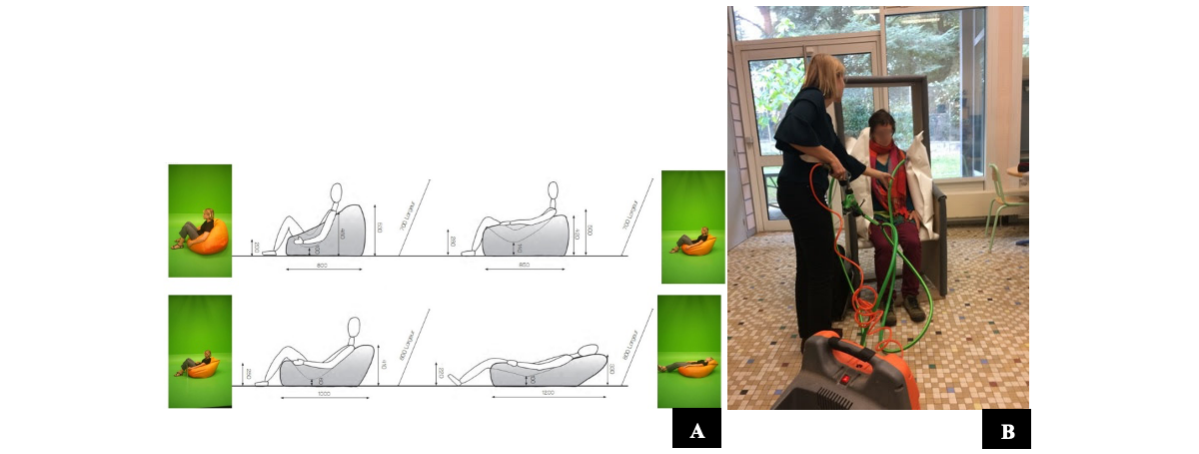
(A) Prototype of the OTO armchair and (B) evaluation of the ergonomics of the chair.

The seat was designed by Alexia Audrain [43], a furniture maker, to address the needs of individuals with ASD for DPT (Figure 1). The project was carried out in partnership with the medical-educational institute of Blain, France, over a period of 1.5 years. After the review of existing technologies, we chose a sitting position that is natural and relaxing but allows one to be active. During the sessions, it allows the professional to keep an eye on the individual with ASD and facilitate the communication to better understand his needs. To assess the ergonomics of the shape of the chair, we tested several inclinations with a Sacco or a bean bag chair (a large fabric bag filled with polystyrene beans; Figure 2A). This allowed us to test several postures and choose one.

The educators, psychomotrician, and the director of the center identified the requirements of the device and gave feedback during the design process. The prototype consisted of inflatable cushions and was used to understand the amount of pressure required to verify the principle of action required to apply side pressure on the body and define the main technical characteristics (Figure 2B).

The sociomedical team (around 10 professionals) and a convenience sample of 30 children with ASD tested the prototype. The designer observed the behaviors of the professionals and children during the test and asked questions about the experience of using the device.

Based on the test results, different models of a compressive chair were designed and sketched in 3 dimensions to validate the form, the materials, and the colors of the prototype before construction. The first model was made in June 2019 and presented to medical-educational institute professionals, children, and the graduation committee. After this, the model was presented to a general audience and another medical educational institute (specialized educators, speech therapist, occupational therapist, and psychologist), Saint-Jean-de-Boiseau near Nantes, France, and was tested with 5 children. The device was also used with a nonverbal adult (30 y) with ASD.

#### Testing Within the Hospital and Modification

In December 2020, OTO, a squeezing armchair, was presented to and tested by the Tours hospital team before introducing it for use in the hospital. The hospital team gave suggestions to enhance the experience of people with ASD.

New functionalities were added before the seat was installed in a day care hospital:

- Access to pressure measurement in real time for the professional
- Logging of pressure measurement, action used, and timestamp
- Seat operation monitoring and safety protocol in case of failure
- Remote control connected to the outside of the seat

#### Integration in the Ward

The device was transferred to the autism day hospital of the Excellence Center of Neurodevelopmental Disorders in the university hospital of Tours, France, in January 2021. After obtaining consent from parents, 39 children with ASD and ID used the device in everyday care (weekly or biweekly). The initial sessions were filmed, with pressure logs recorded by the device, in order to better understand its use in real life. The improvements made after these sessions include the following: cushions were tailored to the morphology of participants, deflation of the upper and lower cells was dissociated, a control panel was used to set the pressure limit, and the feedback light was deactivated because it was too stimulating for children. The noise accompanying inflation and deflation was reduced.

A second group of modifications were made after 2 months and implemented in March 2021:

- A range of different sizes of back cushion
- New cells with a different design for the upper and lower cells to adjust the squeezing effect
- New valves, pipes, and pump to reduce noise
- New remote design with 4 buttons to reduce air pressure independently in the upper or lower cells, and a light that can be deactivated
- A tablet connected to the seat that lets the professional define the maximum pressure

Between April and November, the seat was used in the hospital and tested for 2 months in services in Rennes, Angoulème, and Grandchamps-des-Fontaines. Based on the request of additional features from professionals in Tours and feedback from the 3 other services, some adjustments were made:

- Enhanced experience: easier on/off process, enhanced pressure measurement
- Noise reduction with valve modification and firmware adjustment
- Control panel enhancement, addition of a second remote
- Safety: continuous monitoring of program execution, error handling, and codification

#### Description of the End Product

**Figure 3:**
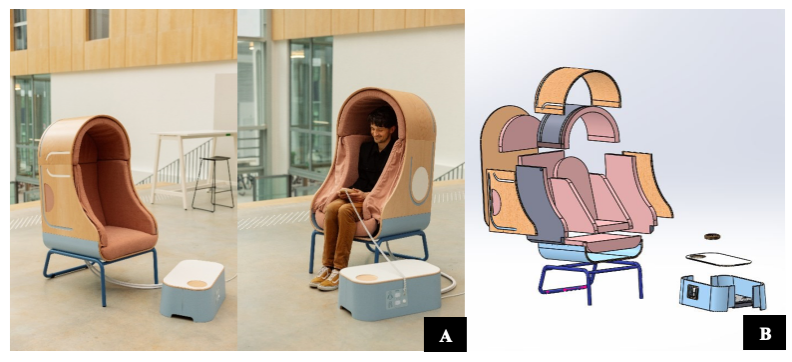
(A) OTO, a compressive armchair to induce deep pressure in children with ASD (final product) and (B) presentation of the different components of the OTO chair. ASD: autism spectrum disorder.

OTO is a squeezing armchair that uses inflatable cells to induce deep pressure on the legs and the trunk (Figure 3; see video [44]). The pressure is progressive, measurable, and homogeneous and can be tailored for each child. Four different inflatable cells allow for modulation by varying pressures on either the shoulders, arm and trunk, or the hip and thighs.

The pressure can be controlled by the children via a remote with simple pictograms, which improves autonomy and predictability for the child. The control panel allows the children to set the maximum pressure level for the upper and lower cells. The maximum default pressure is 60 mm Hg for the upper cells and 80 mm Hg for the lower cells. This corresponds to the pressure a swimmer perceives 1 m under water. The seat records the use of the device with accompanying time logs.

The sitting position makes the device less bulky and allows more freedom of movement for the children, who do not need to lay down as they would in a hug machine. It allows the child to easily leave the armchair if uncomfortable. The footrest can be used to rest the legs, as a step for smaller children to enhance stability, and allow the health care provider to have the same height as the children and to maintain eye contact.

Pastel colors were used to limit sensory stimulation. Edges were avoided to make the armchair safer and more attractive. The device was developed to look like a cocoon to induce a feeling of privacy, to limit the stigmatization of its use, and to limit outside noise or light stimulation. The structure is made from beech wood with a metallic structure for the base. All cloth is removable and washable.

### Measures of Clinical Impressions

Clinical impressions were collected via a logbook with visual analog scales and free report in a day care hospital.

### Technology Readiness Level

The technology readiness level allows us to assess the level of maturity of a technology [45].

### Human-Centered Design for Personal Health Tools

The Human-Centered Design for Personal Health Tools (UCD-11, a validated scale based on a systematic review of the design and development processes of 348 personal health tools) was used to assess the design process [46].

### Time of Use

Beyond this design, it is important to assess the time of use of the device to assess acceptability in children and professionals. Here, it was measured by the chair.

### Measure of Usability

Usability is very important when a system is used with children with special needs and should be assessed in real life [47]. The System Usability Scale (SUS) measured how psychometricians, nurses, and educators perceived the usability of the system [48]. The SUS has been used previously in autism and measures perceived usability from the perspective of professionals, rather than patients themselves [49]. It was measured in a day care hospital.

### Feasibility of an Efficacy Study

We used the National Institute of Health and Care Research definition reported in first figure in the paper by Eldridge et al [50].

### Ethical Considerations

As a design study, this type of research is not a clinical research, thus it is not subject to public health code and associated ethical rules, according to French regulations (Code de la santé publique - Article R1121-1; Legifrance).

## Results

### Usage

Recruitment was done between January and July 2023 in the ASD day hospital in Tours. A total of 39 children aged between 3 and 12 years with ASD and ID were included. Four children had to stop using the chair because of difficulties in tolerating noise, being in an enclosed space, or continuing anxiety despite habituation. These children had difficulties in sensory modulation and motor and emotional regulation. The system was adapted to decrease the inflation and deflation sound of the device.

The children used the remote heterogeneously. Some children controlled the remotes by themselves, and others relied on the professional. Different levels of pressure were used with different rhythms of pressure and deflation.

### First Clinical Impression

First clinical impressions suggest an increase in pleasure and body relaxation, a decrease in anxiety, better postural stability, and better social contact (gaze and touch). When children came back to the group, it appeared that their attention and emotional regulation had improved.

However, in this population, there was marked intra- and interperson variability. A formal evaluation in a clinical trial with a larger sample and controlled procedures is required to formally assess the efficacy of the device.

### Technology Readiness

The technology readiness level was between 8 and 9, meaning that the system can be used for several patients. Minor bugs and user-interface issues with the control panel need to be rectified.

### Human-Centered Design for Personal Health Tools

We used several methods following the recommendations of UCD-11 (Textbox 1).

**Application of the Human-Centered Design for Personal Health Tools (UCD-11) during the design of the OTO chair.**

**1. Were potential end users (eg, patients, caregivers, family and friends, and surrogates) involved in any steps to help understand users (eg, who they are and in what context might they use the tool) and their needs?**

- We performed (1) ethnographic observation of existing practices; (2) informal needs assessment; (3) contextual inquiry, (4) literature review summarized here, and (5) a training webinar to discuss with individuals with autism spectrum disorder (ASD) their sensory issues, needs, and how the OTO chair could be used in everyday life. A protocol to assess the efficacy was developed through a focus group led by sociologists. Several scales to measure outcomes were suggested during this focus group.

**2. Were potential end users involved in any steps of designing, developing, and/or refining a prototype?**

- After ethnographic observation of existing practices, psychometricians gave feedback after sessions with patients to help develop and refine the prototype.

**3. Were potential end users involved in any steps intended to evaluate prototypes or a final version of the tool?**

- We assessed usability among professionals (psychometricians, nurses, and educators) involved in guiding children during use of the OTO chair. The professionals gave feedback after the use of prototypes and again after the use of the final product.

**4. Were potential end users asked their opinions of the tool in any way?**

- We are finalizing focus groups with children, parents, and professionals (reported elsewhere) to assess the sensory issues of children with ASD and the use of different tools and techniques to tackle sensory issues in ASD.

**5. Were potential end users observed using the tool in any way?**

- The sessions were filmed to allow the designer to tailor the design of the chair to the needs of the children.

**6. Did the development process have 3 or more iterative cycles?**

- Four iterations were done.

**7. Were changes between iterative cycles explicitly reported in any way?**

- Major changes are reported in this paper.

**8. Were health professionals asked their opinion of the tool at any point?**

- Child and adolescent psychiatrists, psychomotricians, nurses, and educators provided feedback. These professionals are the most likely to use the OTO chair with children. We gathered feedback on the usability of the chair and observed them using the tool.

**9. Were health professionals consulted before the first prototype was developed?**

- Ethnographic evaluations were carried out with professionals.

**10. Were health professionals consulted between initial and final prototypes?**

- After the prototype was developed, health professionals gave feedback, which was used to finalize the design of the product.

**11. Was an expert panel involved?**

- The armchair received several prizes from several committees:
  - The Canopé (€5000, being US $5560), a national innovation competition organized by Forinvest and the Superior School of Wood, specialized in wood technology.
  - The St Pierre Foundation health innovation prize (€25,000, being US $28,000). The St Pierre Foundation specializes in children’s health, and the award is decided by a panel of medical professionals.
  - James Dyson award for design. Awarded by the James Dyson Foundation and decided by a panel of engineers.
  - Startup and innovation day prize, 2022. This prize recognizes innovative startups.
  - Crédit Mutuel 4S Semeur d’innovation 2023.
  - Caisse d’épargne mon projet innovant 2021.
  - French Tech Tremplin for innovative companies. Launched by people underrepresented in the tech industry.
  - Handitech trophy, awarded by the French Ministry of Health and French Ministry of Digital Technology.

### Time of Use

In a day care hospital, the first sessions were habituation sessions of around 5 minutes. The system was used for 3-20 minutes weekly or biweekly, with 272 hours of total use in the ward. The system was always used with a therapist and never alone and planned around the schedule of the children.

### Usability of the Product

The SUS was carried out with 9 professionals (3 psychomotricians, 3 nurses, and 3 educators) in July 2022 in a day care hospital. A mean score of 81 out of 100 was obtained (indicating between good and excellent usability), corresponding to a B score in a scale from A (best) to F (worst) [51].

### Feasibility of an Efficacy Study

The feasibility of an efficacy study was determined as follows:

1. SD of the outcome measure, which is needed in some cases to estimate the sample size: We could not measure clinical data because of regulations; thus, it is not possible to estimate the sample size. The literature review found that results from a similar device tested on 12 patients supported our clinical impression.
2. Willingness of participants to be randomized: During a focus group in October 2022, parents of children using the chair and 1 autistic adult confirmed their interest in the device and their willingness to participate in an efficacy trial.
3. Willingness of clinicians to recruit participants: Clinicians in a day care hospital and 5 other centers expressed willingness to participate in an efficacy study.
4. Number of eligible patients: With a prevalence of 1%, ASD is quite frequently diagnosed. A lot of children with ASD also have sensory issues. The number of eligible patients seems large enough to conduct an efficacy study.
5. Characteristics of the proposed outcome measure: In the same focus group, in October 2022, the suggested outcome measure (Child Behavior Checklist) was not considered suitable as it was not considered specific enough. Other suggested outcome measures were considered relevant.
6. Adherence and compliance rates: There was good compliance with the device, with only a few dropouts during the early phase of design when the system was too loud for some participants. Most of the questionnaires are already used in clinical practice; others seemed acceptable by the focus group.
7. Availability of data needed: Most of the children in the center have a proper diagnosis. If recruitment is done in other centers, the absence of use of Autism Diagnostic Interview-Revised (ADI-R) and Autism Diagnostic Observation Schedule (ADOS) and poor experience of clinical research could be a limitation.
8. Time needed to collect and analyze data: Getting all the administrative authorization for a medical device in at-risk population (children with ID) and the prospective organization of an efficacy study, planned to run over 12 weeks, may make time management complicated.

## Discussion

### Overview

The process of development of an ergonomic compressive chair to induce deep pressure in children with ASD is described. The design was user centered according to the methodology of Witteman et al [46]. The system is considered usable by professionals according to the SUS.

### User-Centeredness

This device was accepted by the clinicians and patients and their family. The system was primarily used by psychomotor and occupational therapists, but use by nurses and educators was also possible. It did not require the support of a technician. The goal was to increase the acceptability of the device and autonomy of the participant, as well as decrease the stigmatization associated with ASD and its care.

### Armchair Use, Child Profile, and Time of Use

According to the experience of the psychomotricians and analysis of video footage by an independent clinician, usability was better for older children (>8 y). There were no side effects reported. Children could leave the armchair easily. If they experienced discomfort, the therapist was able to deflate the cushion.

The system was used in a small room with limited visual and auditory stimulation. Sometimes, professionals suggested children to use a neck cushion to improve relaxation.

Time of use shows that the device was included in everyday care and suggests that it would be routinely adopted in practice.

### Future Plans

#### Acceptability

Ongoing focus groups and simulations with children, parents, and professionals will examine perspectives on sensory issues in ASD and the acceptability of different devices proposed for DPT and sensory therapy. This will provide more formal data on the perspectives of different users on the sensory peculiarities and needs of people with ASD, as well as solutions and the role of this compressive armchair as a therapeutic approach.

#### Randomized Controlled Trials

This device will enable further evaluation of DPT. In future, we plan to properly characterize and report the data of the individuals including precise diagnostic information (ADI-R and ADOS), their sensory profile and proprioception deficits [16], score on the Echelle des Particularités Sensori-psychomotrices dans l’Autisme (EPSA) scale [52], and underlying pathophysiological processes (eg, heart rate variability and electrodermal activity) using a wearable monitoring device to measure physiological data. To improve acceptability in children with most anxiety, it seems that the presentation of the device should be progressive.

Recruitment for a controlled efficacy study of DPT in ASD seems promising. We have received requests from teams of the original study to be involved in testing the device and expressing willingness to be involved in an efficacy study. Despite complexity of administrative authorizations and time management of a prospective study, such a study seems feasible.

### Limitations

#### Usability Testing

There is a consensus on the need to improve usability of devices in ASD but not on the methods used to measure usability [47]. The SUS is widely used to measure usability, but it can be difficult to use it in individuals with ASD. Thus, it can be amended for use in persons with autism or be filled in by the professional accompanying them [47,53,54]. Usually, the SUS questionnaire is filled in by the person using the system. Here, and in previous studies such as Zhong et al [4], usability was reported by the therapist. Amended versions of current usability tests or the development of alternative means of assessment would improve the assessment of usability.

We think that the time of use and feedback from experts (UCD-11, item 11) reported here and the simulations and focus groups with patients and their parents that we plan to report later are complementary methods that are in favor of a good usability.

#### Measures of Efficacy

This study does not allow any firm conclusions to be drawn about the efficacy of the device in reducing anxiety in ASD. However, this study showed that an efficacy study is feasible [50]. In future efficacy studies, it would be important to report the precise clinical profiles of children and their pressure needs. Because of the design and preliminary nature of the study, French regulations do not allow the reporting of clinical data.

### Implication for Occupational Therapy Practice

This device has the potential to facilitate the design of well-conducted studies to better understand the rationale behind using and the efficacy of DPT in ASD.

### Conclusions

We describe the design process, end product, and user feedback after the use of a compressive chair to conduct DPT in children with ASD. This device would allow the children or adults with ASD to better control the pressure and facilitate high-quality studies to understand the rationale behind using (role of proprioception) and the efficacy of DPT in reducing anxiety in children with ASD.

## Abbreviations

ADI-R: Autism Diagnostic Interview-Revised
ADOS: Autism Diagnostic Observation Schedule
ASD: autism spectrum disorder
DPT: deep pressure therapy
EPSA: Echelle des Particularités Sensori-psychomotrices dans l’Autisme
ID: intellectual deficiency
SUS: System Usability Scale
UCD-11: Human-Centered Design for Personal Health Tools

## References

[1] (2013). Diagnostic and Statistical Manual of Mental Disorders (DSM-5®).

[2] Kojovic N, Ben Hadid L, Franchini M, Schaer M (2019). Sensory processing issues and their association with social difficulties in children with autism spectrum disorders. J Clin Med.

[3] Asmika A, Oktafiani LDA, Kusworini K, Sujuti H, Andarini S (2018). Autistic children are more responsive to tactile sensory stimulus. Iran J Child Neurol.

[4] Zhong X, Wang L, Xu L, Lian J, Chen J, Gong X, Shao Y (2023). Disturbance of skin sensation and autism spectrum disorder: a bidirectional Mendelian randomization study. Brain Behav.

[5] Bonnet-Brilhault F, Tuller L, Prévost P, Malvy J, Zebib R, Ferré S, Dos Santos C, Roux S, Houy-Durand E, Magné R, Mofid Y, Latinus M, Wardak C, Aguillon-Hernandez N, Batty M, Gomot M (2018). A strategic plan to identify key neurophysiological mechanisms and brain circuits in autism. J Chem Neuroanat.

[6] Guinchat V, Vlamynck E, Diaz L, Chambon C, Pouzenc J, Cravero C, Baeza-Velasco C, Hamonet C, Xavier J, Cohen D (2020). Compressive garments in individuals with autism and severe proprioceptive dysfunction: a retrospective exploratory case series. Children (Basel).

[7] Blanche EI, Reinoso G, Chang MC, Bodison S (2012). Proprioceptive processing difficulties among children with autism spectrum disorders and developmental disabilities. Am J Occup Ther.

[8] Morris SL, Foster C J, Parsons R, Falkmer M, Falkmer T, Rosalie S M (2015). Differences in the use of vision and proprioception for postural control in autism spectrum disorder. Neuroscience.

[9] Fuentes C, Mostofsky SH, Bastian AJ (2011). No proprioceptive deficits in autism despite movement-related sensory and execution impairments. J Autism Dev Disord.

[10] Robertson CE, Baron-Cohen S (2017). Sensory perception in autism. Nat Rev Neurosci.

[11] Case-Smith J, Weaver LL, Fristad MA (2015). A systematic review of sensory processing interventions for children with autism spectrum disorders. Autism.

[12] Watling R, Hauer Sarah (2015). Effectiveness of ayres sensory integration® and sensory-based interventions for people with autism spectrum disorder: a systematic review. Am J Occup Ther.

[13] Lancaster S, Zachry A, Duck A, Harris A, Page E, Sanders J (2016). Delivery of the Wilbarger protocol: a survey of pediatric occupational therapy practitioners. J Occup Ther Sch Early Interv.

[14] Watling R, Miller Kuhaneck H, Parham LD, Schaaf R (2018). Occupational Therapy Practice Guidelines for Children and Youth with Challenges in Sensory Integration and Sensory Processing.

[15] Green VA, Pituch KA, Itchon J, Choi A, O'Reilly M, Sigafoos J (2006). Internet survey of treatments used by parents of children with autism. Res Dev Disabil.

[16] Peña M, Ng Y, Ripat J, Anagnostou E (2021). Brief report: parent perspectives on sensory-based interventions for children with autism spectrum disorder. J Autism Dev Disord.

[17] Charleson JL (2024). Effectiveness of weighted blankets as an intervention for sleep problems in children with autism [Thesis]. University of Canterbury.

[18] Gringras P, Green D, Wright B, Rush C, Sparrowhawk M, Pratt K, Allgar V, Hooke N, Moore D, Zaiwalla Z, Wiggs L (2014). Weighted blankets and sleep in autistic children--a randomized controlled trial. Pediatrics.

[19] Eron K, Kohnert L, Watters A, Logan C, Weisner-Rose M, Mehler PS (2020). Weighted blanket use: a systematic review. Am J Occup Ther.

[20] Bolic Baric V, Skuthälla S, Pettersson M, Gustafsson PA, Kjellberg A (2023). The effectiveness of weighted blankets on sleep and everyday activities - a retrospective follow-up study of children and adults with attention deficit hyperactivity disorder and/or autism spectrum disorder. Scand J Occup Ther.

[21] Ekholm B, Spulber S, Adler M (2020). A randomized controlled study of weighted chain blankets for insomnia in psychiatric disorders. J Clin Sleep Med.

[22] Steingrímsson S, Odéus E, Cederlund M, Franzén S, Helgesson C, Nyström K, Sondell J, Opheim A (2022). Weighted blanket and sleep medication use among adults with psychiatric diagnosis - a population-based register study. Nord J Psychiatry.

[23] Mullen B, Champagne T, Krishnamurty S, Dickson D, Gao Rx (2008). Exploring the safety and therapeutic effects of deep pressure stimulation using a weighted blanket. Occup Ther Ment Health.

[24] Becklund AL, Rapp-McCall L, Nudo J (2021). Using weighted blankets in an inpatient mental health hospital to decrease anxiety. J Integr Med.

[25] Biswas TT, Infirri RS, Hagman S, Berglin L (2018). An assistive sleeping bag for children with autism spectrum disorder. Fash Text.

[26] Opsommer E, Dubois J, Bangerter G, Panchaud R, Martin D, Skuza K (2016). Therapeutic body wraps in Swiss public adult acute inpatient wards. a retrospective descriptive cohort study. J Psychiatr Ment Health Nurs.

[27] Delion P, Labreuche J, Deplanque D, Cohen D, Duhamel A, Lallié C, Ravary M, Goeb JL, Medjkane F, Xavier J (2018). Therapeutic body wraps (TBW) for treatment of severe injurious behaviour in children with autism spectrum disorder (ASD): A 3-month randomized controlled feasibility study. PLoS One.

[28] Amaral D, Rogers SJ, Baron-Cohen S, Bourgeron T, Caffo E, Fombonne E, Fuentes J, Howlin P, Rutter M, Klin A, Volkmar F, Lord C, Minshew N, Nardocci F, Rizzolatti G, Russo S, Scifo R, van der Gaag RJ (2011). Against le packing: a consensus statement. J Am Acad Child Adolesc Psychiatry.

[29] Chamak B (2020). Packing : quand des parents témoignent [Article in French]. Neuropsychiatrie de l'Enfance et de l'Adolescence.

[30] Duvall JC, Schleif N, Dunne LE, Holschuh B (2016). Active "hugging" vest for deep touch pressure therapy.

[31] Duvall JC, Schleif N, Dunne LE, Holschuh B (2019). Dynamic compression garments for sensory processing disorder treatment using integrated active materials. J Med Device.

[32] Reynolds S, Lane SJ, Mullen B (2015). Effects of deep pressure stimulation on physiological arousal. Am J Occup Ther.

[33] Watkins N, Sparling E (2014). The effectiveness of the snug vest on stereotypic behaviors in children diagnosed with an autism spectrum disorder. Behav Modif.

[34] Bestbier L, Williams TI (2017). ). The immediate effects of deep pressure on young people with autism and severe intellectual difficulties: demonstrating individual differences. Occup Ther Int.

[35] Krauss KE (1987). The effects of deep pressure touch on anxiety. Am J Occup Ther.

[36] Edelson SM, Edelson M G, Kerr D C, Grandin T (1999). Behavioral and physiological effects of deep pressure on children with autism: a pilot study evaluating the efficacy of grandin's hug machine. Am J Occup Ther.

[37] Minoura M, Tani I, Ishii T, Gunji YP (2021). Squeezed and released self: using a squeeze machine to degrade the peri-personal space (PPS) boundary. Psychol Conscious.

[38] Lo JS, Huang SC (2018). Creative design of sitting hug machine in the treatment of students with autism. MATEC Web Conf.

[39] Afif IY, Maula MI, Aliyafi MB, Aji AL, Winarni TI, Jamari J (2021). Design of hug machine portable seat for autistic children in public transport application. IOP Conf Ser: Mater Sci Eng.

[40] Maula MI, Aji Al, Aliyafi Mb, Afif Iy, Ammarullah Mi, Winarni Ti, Jamari J (2021). The subjective comfort test of autism hug machine portable seat. J Intellect Disabl Diagn Treat.

[41] Grandin T (1992). Calming effects of deep touch pressure in patients with autistic disorder, college students, and animals. J Child Adolesc Psychopharmacol.

[42] Lane SJ, Mailloux Z, Schoen S, Bundy A, May-Benson TA, Parham LD, Smith Roley S, Schaaf RC (2019). Neural Foundations of Ayres Sensory Integration®. Brain Sci.

[43] OTO chair, Alexia Audrain. LABAA.

[44] OTO chair demonstration, Alexia Audrain. LABAA.

[45] (2006). Defense Acquisition Guidebook.

[46] Witteman H, Vaisson G, Provencher T, Dansokho SC, Colquhoun H, Dugas M, Fagerlin A, Giguere A, Haslett L, Hoffman A, Ivers N, Legare F, Stacey D, Trottier ME, Volk R, Renaud JS (2019). Development and validation of UCD-11: an 11-item measure of user-centered design for patient-centered tools. OSF Preprints.

[47] Aguiar YPC, Galy E, Godde A, Trémaud M, Tardif C (2020). AutismGuide: a usability guidelines to design software solutions for users with autism spectrum disorder. Behav Inform Technol.

[48] Brooke J (1996). SUS—a quick and dirty usability scale. Usability Evaluation in Industry.

[49] Weiss PL, Gal E, Zancanaro M, Giusti L, Cobb S, Millen L, Hawkins T, Glover T, Sanassy D (2011). Usability of technology supported social competence training for children on the autism spectrum.

[50] Eldridge SM, Lancaster GA, Campbell MJ, Thabane L, Hopewell S, Coleman CL, Bond CM (2016). Defining feasibility and pilot studies in preparation for randomised controlled trials: development of a conceptual framework. PLoS One.

[51] Bangor A, Kortum P, Miller J (2009). Determining what individual SUS scores mean: adding an adjective rating scale. J Usability Stud.

[52] Le Menn-Tripi C, Vachaud A, Defas N, Malvy J, Roux S, Bonnet-Brilhault F (2019). L’évaluation sensori-psychomotrice dans l’autisme : un nouvel outil d’aide au diagnostic fonctionnel sensory-psychomotor evaluation in autism: a new tool for functional diagnosis. L'Encéphale.

[53] Gentile V, Adjorlu A, Serafin S, Rocchesso D, Sorce S (2019). Touch or touchless?: evaluating usability of interactive displays for persons with autistic spectrum disorders.

[54] Parish-Morris J, Solórzano R, Ravindran V, Sazawal V, Turnacioglu S, Zitter A, Miller JS, Mccleery JP (2018). Immersive virtual reality to improve police interaction skills in adolescents and adults with autism spectrum disorder: preliminary results of a phase i feasibility and safety trial. Annu Rev CyberTherapy Telemed.

